# Application of LiMgPO_4_ crystal for proton beam quality control in radiotherapy

**DOI:** 10.1093/rpd/ncac214

**Published:** 2023-10-11

**Authors:** Damian Wróbel, Tomasz Kowalski, Sebastian Kusyk, Barbara Marczewska, Tomasz Nowak, Paweł Olko, Jan Swakoń

**Affiliations:** Institute of Nuclear Physics Polish Academy of Sciences, PL-31342 Krakow, Poland; Institute of Nuclear Physics Polish Academy of Sciences, PL-31342 Krakow, Poland; Institute of Nuclear Physics Polish Academy of Sciences, PL-31342 Krakow, Poland; Institute of Nuclear Physics Polish Academy of Sciences, PL-31342 Krakow, Poland; Institute of Nuclear Physics Polish Academy of Sciences, PL-31342 Krakow, Poland; Institute of Nuclear Physics Polish Academy of Sciences, PL-31342 Krakow, Poland; Institute of Nuclear Physics Polish Academy of Sciences, PL-31342 Krakow, Poland

## Abstract

The radioluminescence (RL) emitted by LiMgPO_4_ detector under proton beam irradiation was investigated in real time at the radiotherapy facility in the Institute of Nuclear Physics Polish Academy of Sciences in Krakow. The facility uses protons accelerated by the AIC-144 isochronous cyclotron up to the energy of 60 MeV. The measurements of RL were carried out using a remote optical fiber device with a luminophore detector and photomultiplier located at opposite ends of the optical fiber. A thin slice of LiMgPO_4_ doped with Tm (1.2 mol%) crystal was exposed to the proton beam. The tested detector allowed for the measurement of proton beam current, flux fluence and determination of proton beam time structure parameters. The investigation of LiMgPO_4_ crystal showed its high sensitivity, fast reaction time to irradiation and possibility of application as the detector for control of proton beam parameters.

## Introduction

Proton radiotherapy is increasingly becoming popular and more accessible therapy used over the world. The AIC-144 cyclotron installed at Institute of Nuclear Physics Polish Academy of Sciences (IFJ PAN)^(1–3)^ became a proton source for the research and development of new dosimetry methods for radiotherapy.

One of the dose measurement methods currently being under development is real-time dosimetry based on the measurement of radioluminescence (RL) emitted by a detector in form of a piece of luminophore crystal. RL relies on the emission of light under irradiation, which accompanies transitions between energy levels in a luminophore crystal. It occurs spontaneously during exposure to ionizing radiation, and the measured light intensity is proportional to dose rate. Fiber optically coupled luminescence detectors provide a promising supplement to other dosimetry methods by offering the capability of real-time *in vivo* dose monitoring with high time and spatial resolution. The fundamental requirements for detectors used for this purpose are their high sensitivity to radiation and small size, wherein the large signal loss in time after irradiation is not an obstacle in this application.

This real-time measurement method proposed several years ago was based on the luminescence of crystalline aluminum oxide doped with carbon (Al_2_O_3_:C) located at the end of the long optical fiber connected to the sensitive light detector (a photomultiplier (PMT)) for signal recording and a laser for detector stimulation^(4)^. The research has been concentrated on commercially available Al_2_O_3_:C^(5, 6)^ as well as BeO^(7, 8)^.

High sensitivity to the ionizing radiation and the fast loss of the signal in time after irradiation are the features of LiMgPO_4_ (LMP). It is considered as one of the new promising dosimetric materials. Several groups work on the development of LiMgPO_4_, which is usually doped with Tb and B and produced in a form of powder^(9–13)^. Recently, investigations have been also performed on crystals grown from LMP powder by the micro-pulling down method (MPD)^(14–23)^ or thin foils^(24, 25)^.

Lately, the LMP has been tested for use as a detector in a PORTOS measuring device that was developed by our team^(23)^. This method is based on the real-time measurement of RL emitted by a small luminophore placed on the end of optical fiber connected to a PMT. The tests of the device performed in Cs-137 and Co-60 gamma radiation fields showed that PORTOS is a fast-reacting device with a wide linear dose response^(23)^. Therefore, the next step is to use it for testing in the proton beam.

The study aimed to investigate the properties of LiMgPO_4_ crystal doped with Tm under proton beam in regard to its application as a detector for proton beam real-time monitoring and control of parameters such as time profile of beam cycles, duration of the pulse and linearity in a wide range of beam currents (flux) at the AIC-144 cyclotron.

## Methods

A small portable remote device called PORTOS^(23)^ has been used in this experiment. The PORTOS optical system consists of PMT Hamamatsu H10682-210 located at one end of a 15-m quartz optical fiber, at the other end of which detector, in the form of a slice of a crystal (thickness of 1 mm), was placed. The set of optical filters was situated in the panels between the PMT and the detector. The PORTOS system possesses also a dichroic mirror, intended for OSL measurement in the future, for changing the direction of the laser beam which can be easily attached to the system. The set of optical filters was selected based on previously performed spectral investigations of the RL light emitted by Tm-doped LMP crystals^(22)^. Finally, a short-pass filter transmitting wavelengths between 250 and 475 nm (Edmunds Optics) and two 405/150 nm (transmission between 330 and 480 nm) BrightLine single-band bandpass filters (Semrock) were applied. The PORTOS system is controlled by software enabling the signal recording with different sampling times and for the measurement duration up to some hours.

For this experiment, a slice (diameter of 2 mm and 1 mm in thick) of a LiMgPO_4_:Tm (1.2 mol%) crystal was used as a detector placed at the end of the optical fiber and was protected from light with a plastic capsule. The LiMgPO_4_ crystals were grown at IFJ PAN by MPD^(14, 15)^ in the forms of rods of ~2 mm in diameter and length of ~60 mm. The doping took place at the stage of preparing the feed material before the crystal growth. The crystal was cut into slices using diamond wire saw.

Irradiation was performed with a 60-MeV proton beam produced by the AIC-144 cyclotron. In this experiment, the detector has been installed in the proton beam inside the protective cap on the end of a 15-m optical fiber, which transmitted the optical signal to the PMT tube of the PORTOS system in the control room.

RL signal recorded by the PMT comes not only from the detector but also from the part of the optical fiber that is under the influence of radiation and emits its own signal called ‘stem’. To separate the signal from the detector itself from the device signal, measurements of stem were carried out before each actual measurement using the optical fiber without a detector. The stem value was subtracted from the total RL in linearity of response investigations.

## Results

### Time structure determination

The proton beam produced by the AIC-144 cyclotron has a micro- and macrostructure. The proton beam microstructure is related to the frequency of the AIC-144 HV generator, the micro-impulses are generated at a frequency of 26.26 MHz. Micro-pulses are grouped into packets with a length of ~0.5 ms with a frequency of 50 Hz. The first part of the experiment has been to determine the time structure of the proton beam.

**Figure 1:**
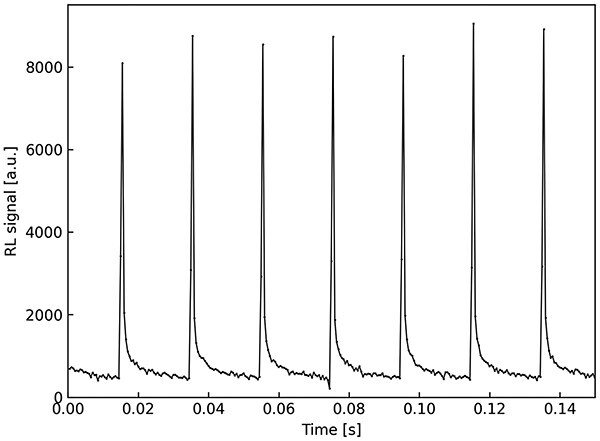
Time profile of 60 MeV proton beam measured as RL signal of LiMgPO_4_:Tm (1.2 mol%) detector under proton beam; macro-pulses are separated by 20 ms pause, sampling time 0.5 ms.

Figure 1 presents time profile of 60-MeV proton beam measured as RL signal from LiMgPO_4_:Tm (1.2 mol%). Crystal detector was mounted in the ax of 40 mm broad beam close to isocentre of facility and was connected to PORTOS device to collect data. Sampling time has been set as 0.5 ms which allows to observe the macro-pulses of the beam separated by 20-ms pause.

**Figure 2:**
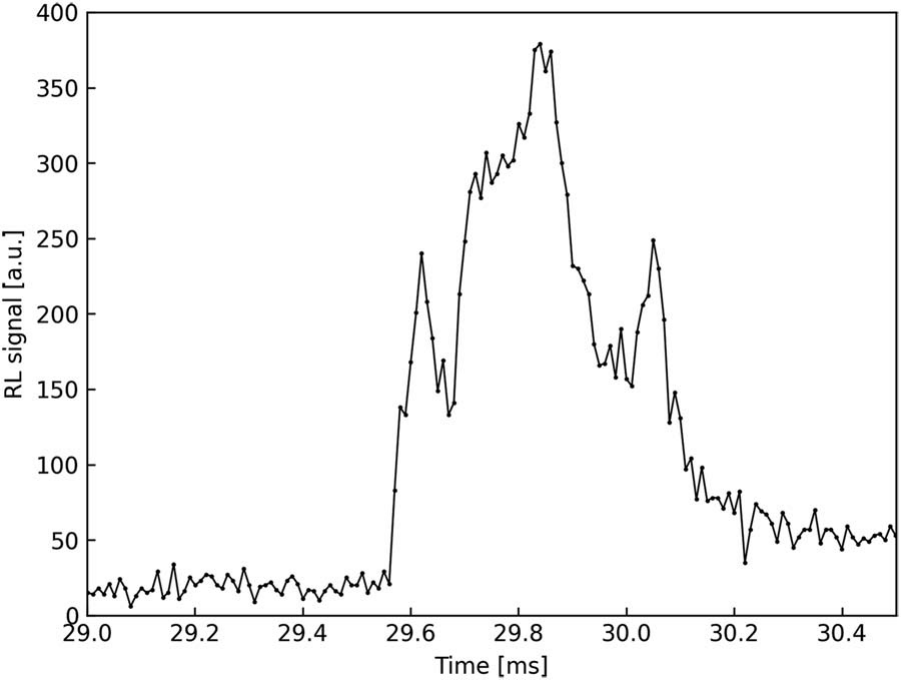
Macro-pulse structure measured as RL signal of LiMgPO_4_:Tm (1.2 mol%) detector under proton beam, sampling time 10 μs.

Further investigations concerned macro-pulse duration and structure. Sampling time of PORTOS system has been reduced to 10 μs and data were recorded for 1.5 ms. Such a sampling rate allowed to register the internal structure of the macro-pulse, which also made it possible to determine the length of a single macro-pulse. Figure 2 illustrates an example of macro-pulse structure measured with 10-μs sampling time. The analysis of macro-pulses showed that the length of the macro-pulse is constant in time and approximately equals 0.5 ms. The registered shapes of individual macro-impulse indicate that the flux of protons inside the macro-impulse is not constant but is subject to significant fluctuations.

### Lateral beam profile measurements

**Figure 3:**
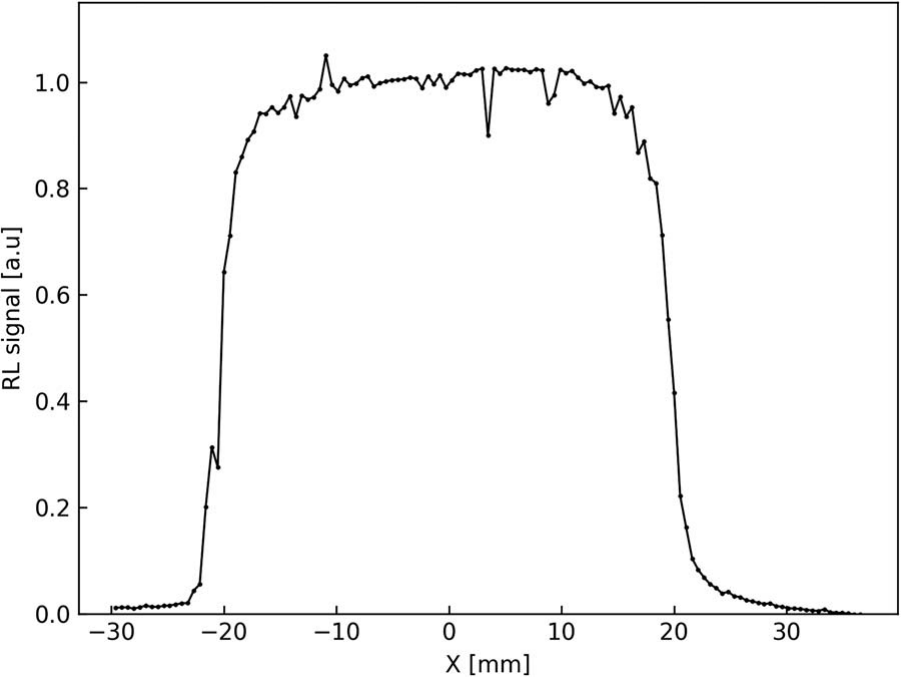
Horizontal profile of the proton beam measured as RL signal of LiMgPO_4_:Tm (1.2 mol%) detector under proton beam, sampling time 0.4 s.

The LMP detector can be used to determine spatial parameters of the proton beam, such as depth dose distribution, and to record lateral dose distributions or fluence distributions. As an example, the measurement of the lateral fluence distribution of the beam is given for a beam with a width of 40 mm. During the measurements, the LMP detector attached to the X-scanner was moved across the field along the horizontal ax of the proton beam with a diameter of 40 mm. The sampling time was set at 0.4 s, and the scanning time between the measurement points was negligible. PORTOS’s software gives information only about the signal and time of receiving that signal, but establishing scanner velocity allows to recalculate time units into length units. Figure 3 demonstrates the horizontal proton beam profile. The data were corrected by a linear function to eliminate accretion in time effect.

### Linearity of the response

RL signal of LiMgPO_4_:Tm (1.2 mol%) detector has a linear dependency on proton beam flux. The linearity of the RL response of the LMP detector has been checked by changing the output current from the cyclotron in the range from 1 nA to 50 nA, which allowed to check the linearity of the response in the range from 9.2E6 to 4.6E8 p/(cm^2^s).

**Figure 4:**
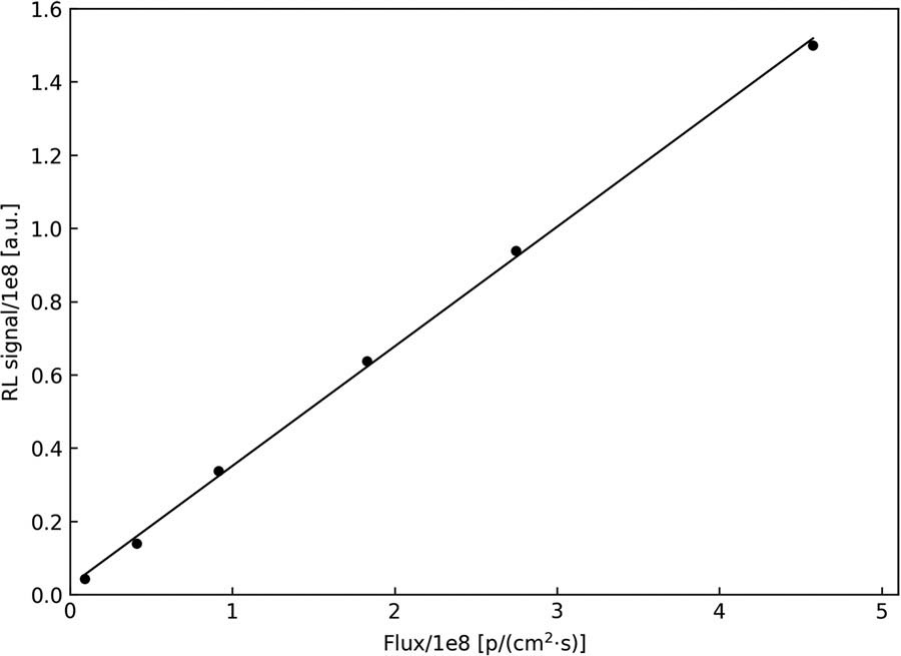
Total RL signal of LiMgPO_4_:Tm (1.2 mol%) detector under proton beam measured in 30 s with sampling time 1 s as a function of proton beam flux.

Figure 4 shows measurement points calculated as the sum of 30 points (reduced by analogically calculated stem value) with a fitted linear curve *y* = 0.326*x* + 0.026. The coefficient of determination equals 0.999, which confirms the linearity between the RL signal from the crystal and the flux of proton beam.

## Conclusions

The LiMgPO_4_ crystal doped with Tm (1.2 mol%) applied as a detector in PORTOS device can be successfully used for proton beam diagnostics and selected beam quality assurance measurements. Measurements with a sampling time of 0.5 ms can be used to determine the macro-pulse time structure of the beam delivered from the AIC-144 cyclotron. Increasing sampling frequency is needed to see the macro-pulse structure. Duration of a pulse was estimated as 0.5 ms with 10-μs sampling time. The internal time structure of a macro-pulse can be registered. As sampling time is limited to 1 μs, it is not possible to record the beam microstructure. The detector can also be used to measure the spatial distribution of a proton beam, e.g. for measurements of lateral fluence distributions. Measurements show the linear response of LiMgPO_4_:Tm (1.2 mol%) signal in the range of proton fluxes typically used at this irradiation facility. Investigation showed that LiMgPO_4_:Tm (1.2 mol%) crystal with PORTOS device can be an efficient tool in quality control in radiotherapy.
